# Facilitating the development of controlled vocabularies for metabolomics technologies with text mining

**DOI:** 10.1186/1471-2105-9-S5-S5

**Published:** 2008-04-29

**Authors:** Irena Spasić, Daniel Schober, Susanna-Assunta Sansone, Dietrich Rebholz-Schuhmann, Douglas B Kell, Norman W Paton

**Affiliations:** 1 Manchester Centre for Integrative Systems Biology, The University of Manchester, 131 Princess Street, Manchester, M1 7ND, UK; 2 School of Computer Science, The University of Manchester, Oxford Road, Manchester, M13 9PL, UK; 3 The European Bioinformatics Institute, EMBL Outstation - Hinxton, Wellcome Trust Genome Campus, Cambridge, CB10 1SD, UK; 4 School of Chemistry, The University of Manchester, Oxford Road, Manchester, M13 9PL, UK

## Abstract

**Background:**

Many bioinformatics applications rely on controlled vocabularies or ontologies to consistently interpret and seamlessly integrate information scattered across public resources. Experimental data sets from metabolomics studies need to be integrated with one another, but also with data produced by other types of omics studies in the spirit of systems biology, hence the pressing need for vocabularies and ontologies in metabolomics. However, it is time-consuming and non trivial to construct these resources manually.

**Results:**

We describe a methodology for rapid development of controlled vocabularies, a study originally motivated by the needs for vocabularies describing metabolomics technologies. We present case studies involving two controlled vocabularies (for nuclear magnetic resonance spectroscopy and gas chromatography) whose development is currently underway as part of the Metabolomics Standards Initiative. The initial vocabularies were compiled manually, providing a total of 243 and 152 terms. A total of 5,699 and 2,612 new terms were acquired automatically from the literature. The analysis of the results showed that full-text articles (especially the Materials and Methods sections) are the major source of technology-specific terms as opposed to paper abstracts.

**Conclusions:**

We suggest a text mining method for efficient corpus-based term acquisition as a way of rapidly expanding a set of controlled vocabularies with the terms used in the scientific literature. We adopted an integrative approach, combining relatively generic software and data resources for time- and cost-effective development of a text mining tool for expansion of controlled vocabularies across various domains, as a practical alternative to both manual term collection and tailor-made named entity recognition methods.

## Background

The lack of a suitable means for formally describing the semantic aspects of omics investigations presents challenges to effective information exchange between biologists [1-3]. The inherent imprecision of free-text descriptions of experimental procedures hinders computational approaches to the interpretation of experimental results. Controlled vocabularies and/or ontologies can be used as a means of adding an interpretative annotation layer to the textual information [4-6]. A controlled vocabulary (CV) is a structured set of terms (i.e. linguistic representations of domain-specific concepts [7], and as such a means of conveying scientific and technical information [8]) and definitions agreed by an authority or a community. An ontology includes CV terms to refer to concepts at the linguistic level, but also utilises a richer semantic representation to characterise the ways in which these concepts are related [9]. Many scientific communities, including those operating in the metabolomics domain [10], have started developing ontologies for data annotation [11]. The Metabolomics Standards Initiative (MSI) [12,13] Ontology Working Group (OWG) [14] has been appointed to establish a common semantic framework (i.e. a set of ontologies and their CVs) for metabolomics studies to be used to describe the experimental process consistently, and to ensure meaningful and unambiguous data exchange [15]. While providing a mechanism for coherent and rigorous structuring of domain-specific knowledge, it is necessary for ontologies and CVs in an expanding domain such as metabolomics to be easily extensible. The new knowledge, largely generated by high-throughput screening, is communicated through the biotechnology literature, which can be exploited by text mining (TM) tools to facilitate the process of keeping ontologies and their CVs up to date [6,16]. In this article we describe a TM approach for rapidly expanding a set of CVs maintained by the MSI OWG with terms extracted from the scientific literature, following initial term acquisition from sources such as domain specialists, literature, databases, existing ontologies, etc.

The MSI OWG [17] aims to develop a set of ontologies and CVs in metabolomics as a direct support to the activities of other MSI WGs [15], which are responsible for: Biological Context Metadata, Chemical Analysis, Data Processing and Exchange Formats. The coverage of the domain has been divided in accordance with the typical structure of metabolomics investigations:

• general components (investigation design; sample source, characteristics, treatments and collection; computational analysis), and

• technology-specific components (sample preparation; instrumental analysis; data pre-processing).

The ongoing standardisation endeavours in other omics domains, such as the Human Proteome Organization (HUPO) Proteomics Standards Initiatives (PSI) [18,19], the Microarray Gene Expression Data Society (MGED) [20,21] and other ontology communities under the Open Biomedical Ontologies (OBO) Foundry [22-24] umbrella can largely be re-used to describe the general aspects of metabolomics investigations. Therefore, the MSI OWG has focused initially on the technology-specific components. Further, development activities in this sub-domain have been prioritised according to the pervasiveness of the analytical platforms used.

A range of analytical technologies have been employed in metabolomics studies [25]. Mass spectrometry (MS) is the most widely used analytical technology in metabolomics, as it enables rapid, sensitive and selective qualitative and quantitative analyses with the ability to identify individual metabolites. In particular, the combined chromatography-MS technologies have proven to be highly effective in this respect. Gas chromatography-mass spectrometry (GC-MS) uses GC to separate volatile and thermally stable compounds prior to detection via MS. Similarly, liquid chromatography-mass spectrometry (LC-MS) provides the separation of compounds by LC, which is again followed by MS. On the other hand, nuclear magnetic resonance (NMR) spectroscopy does not require any separation of the compounds prior to analysis, thus providing a non-destructive, high-throughput detection method with minimal sample preparation, which has made it highly popular in metabolomics investigations despite being relatively insensitive in comparison to the MS-based methods.

For MS, the MSI OWG will leverage previous work by the PSI MS Standards WG [26]. For chromatography, which is used in both proteomics and metabolomics, the MSI OWG is closely collaborating with the PSI Sample Processing Ontology WG. Consequently, the technologies the MSI OWG is currently focusing on are NMR and GC. These two technologies are used in this paper to illustrate the effectiveness of the proposed TM approach.

The MSI OWG efforts are divided into two key stages: (1) reaching a consensus on the CVs, and (2) developing the corresponding ontology as part of the Ontology for Biomedical Investigations (OBI, previously FuGO) [27,28]. In this paper, we focus on the first stage. Each CV is compiled in the following three steps:

1. Compilation: An initial CV is created by re-using the existing terminologies from database models (e.g. [29,30]), glossaries, etc. and normalising the terms according to some common naming conventions [31]. The result of this phase is a draft CV encompassing terms of different types: methods, instruments, parameters that can be measured, etc.

2. Expansion: In the highly dynamic metabolomics domain, experts often use non-standardised terms. Therefore, in order to reduce the time and cost of compiling a CV and to strive for its completeness, we use a TM approach to automatically identify additional technology-related terms frequently occurring in the scientific literature.

3. Curation: The CV is discussed within the MSI OWG and is passed on to the practitioners in the relevant metabolomics area for validation in order to ensure the quality and completeness of the proposed CV.

We expect the CVs to evolve in time by reflecting the changes in the domain and the availability of new literature, and therefore steps 2 and 3 should be iterated over in certain time intervals.

## Implementation

**Figure 1:**
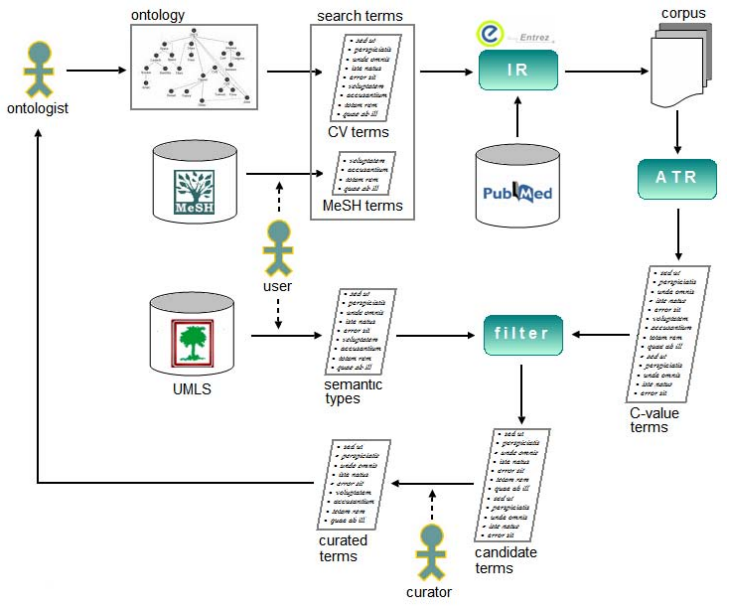
**The flow of data in a TM approach to CV expansion.** The information retrieval module is used to gather a corpus of documents relevant for a given CV from the literature databases. Automatic term recognition is applied against the corpus to extract terms as domain-specific lexical units. Some of the extracted terms not directly related to the CV are filtered out by using the knowledge about typically co-occurring types of terms.

A set of relevant tasks regarding CV term acquisition has been identified, including information retrieval, term recognition and term filtering. Figure 1 summarises the main steps taken in our TM approach to CV expansion. First, the information retrieval module is used to gather documents relevant for a given CV from the literature databases. Once a domain-specific corpus of documents has been assembled, it is searched for potential terms unaccounted for in the initial CV. Automatic term recognition is performed to extract terms as domain-specific lexical units, i.e. the ones that frequently occur in the corpus and bear special meaning in the domain. In order to reduce the number of terms not directly related to a given technology, and therefore not relevant for the given CV, we filter out typically co-occurring types of terms denoting substances, organisms, organs, diseases, etc. In contrast to the considered analytical techniques, these sub-domains have more established CVs, which can be exploited to recognise these terms using a dictionary-based approach [32]. Each of the TM steps is described in more detail in the forthcoming sub-sections.

### Information retrieval

*Information retrieval* (IR) implements the representation, storage and organisation of textual data to enable a user to access relevant pieces of information [33]. Biomedical experts regularly exploit IR to locate relevant information (most often in the form of scientific publications) on the Internet. Apart from general-purpose search engines such as Google™ [34], many IR systems have been designed specifically to query databases of biomedical publications (e.g. [35-39]) such as Medical Literature Analysis and Retrieval System Online (MEDLINE) [40] and PubMed Central (PMC) [41] (henceforth referred to together as *PubMed*), which provide peer-reviewed literature and make it freely accessible in a uniform format. MEDLINE distributes *abstracts* only, while PMC provides *full-text articles*. PubMed is accessible through *Entrez *[42], an integrated retrieval system that provides access to a family of related biomedical databases maintained by the National Center for Biotechnology Information (NCBI).

Documents available in PubMed are indexed by Medical Subject Headings (MeSH) [43] terms (*index terms* are pre-selected to refer to the content of a document [33]). MeSH is a CV consisting of hierarchically organised terms that serve as descriptors to index and annotate documents. This permits direct access to relevant documents at various levels of specificity, thus improving the performance of IR in terms of speed as well as precision and recall. Entrez uses automatic term mapping to match terms against the MeSH hierarchy and to expand a query with (near-)synonyms and subsumed terms. For example, all of the following terms are explicitly listed as terms matching *Magnetic Resonance Spectroscopy* in MeSH:

• *In Vivo NMR Spectroscopy*

• *Magnetic Resonance*

• *MR Spectroscopy*

• *NMR Spectroscopy*

• *NMR Spectroscopy, In Vivo*

• *Nuclear Magnetic Resonance*

• *Spectroscopy, Magnetic Resonance*

• *Spectroscopy, NMR*

• *Spectroscopy, Nuclear Magnetic Resonance*

**Figure 2:**
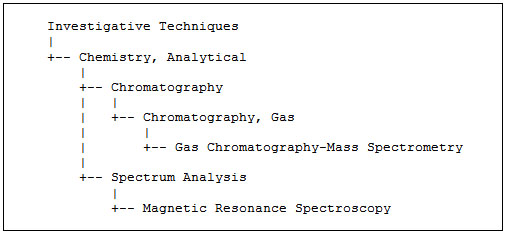
**A sub-tree of the MeSH hierarchy.** We show part of the MeSH hierarchy relevant for the two CVs (i.e. NMR and GC) considered.

Similarly, a query searching for information on *Gas Chromatography* can be expanded automatically to include *Gas Chromatography-Mass Spectrometry* as a more specific term (see figure 2).

While the use of the MeSH for indexing and query expansion in Entrez is undoubtedly useful, these benefits cannot be fully exploited for the particular problem of accessing articles describing research that utilizes some analytical technology. In particular, an analytical technique employed in metabolomics is unlikely to be the main focus of the reported studies. Consequently, the corresponding documents may not necessarily be indexed with technology-related MeSH terms. Further, the abstracts of such articles are more likely to report the actual findings rather than the technology-specific experimental conditions applied. These parameters are usually described in the *Materials and methods* section or as part of the supplementary material. Hence, two points arise when retrieving documents containing information pertinent for analytical techniques deployed in metabolomics studies. First, it is important to search full-text articles as opposed to abstracts only. For this reason we used PMC, which provides access to full-text articles, in addition to MEDLINE, which offers only abstracts. Second, it is necessary to go beyond MeSH terms in query formulation. This problem is alleviated using the following assumption: terms denoting related concepts tend to co-occur within textual documents [44,45]. On this basis, terms from an initially compiled CV can be combined in a search query to retrieve additional documents that describe research that utilises a technology, i.e. the ones that do not necessarily deal with the technology *per se* and thus may not be indexed by technology-related MeSH terms. To achieve this, we index the literature with the CV terms. Each CV term is used to search the literature via Entrez. As a result, each term is mapped to a set of documents it matches. This information is stored in a local database using the following structure described in SQL:

CREATE TABLE index

(

term VARCHAR(200) NOT NULL,

document VARCHAR(50) NOT NULL

);

A cut-off point (this is a configurable parameter; the specific values used in our case studies are reported in the *Results & Discussion* section) is set to remove the non-discriminatory terms, i.e. the ones that return too many documents. These are likely to be broad terms not limited to a specific analytical technique, and consequently introducing unwanted noise in the context of the domain-specific corpus. For example, in the case of the NMR CV, the mean number of abstracts returned was 2,772 with the median being just 0, which is due to the fact that the NMR CV was constructed using a considerable number of terms coming from database schemata. These terms are semi-formal in the sense that they do not necessarily reflect the terminology used in the literature, e.g. *AMIX VIEWER & AMIX-TOOLS* and *JEOL NMR instrument*. On the other extreme, terms returning the maximal number of abstracts (set to 50,000) were: *analysis, characteristic, concentration, Delta, instrument, method, reference, software, states* and *tube*. The following SQL query can be used to identify such terms:

SELECT term, COUNT(document) AS matching_documents

FROM index

GROUP BY term

WHERE matching_documents >= D;

where D is chosen a cut-off point. Having removed such terms from further consideration from the IR point of view, a cut-off point (as before, this is a configurable parameter, and the specific values used in our case studies are reported in the *Results & Discussion* section) is set to remove the documents that do not contain a sufficient number of the CV terms. The following SQL query can be used to identify such documents:

SELECT document, COUNT(term) AS matching_terms

FROM index

GROUP BY document

WHERE matching_terms <= T;

where T is chosen a cut-off point. For example, some of the documents with the highest number of matching terms from the NMR CV were [46-48].

The IR module based on the methods described above is encoded in Java. The Java application takes advantage of E-Utilities [42], a web service which enables the users to run Entrez queries and download data using their own applications. The information gathered about terms, documents and their relations is stored in a local database (DB) hosted on a PostgreSQL [49] system. By storing the mappings between terms and documents, the querying ability of the DB management system can be combined with that of Entrez. The local DB is also accessible via Java applications (using the JDBC protocol – a standard SQL DB access interface). Hence, all our implemented IR modules can be incorporated into customised workflows [50].

### Term recognition

In the literature dealing with terminology issues, a term is intuitively defined as a phrase (typically a noun phrase [7,51]): (1) frequently occurring in texts restricted to a specific domain, and (2) having a special meaning in the given domain [52]. Bearing in mind the potentially unlimited number of different domains and the dynamic nature of newly emerging ones (many of which expand rapidly together with the corresponding terminologies, as is the case in metabolomics), the need for efficient term recognition becomes apparent. Manual term recognition approaches are time-consuming, labour-intensive and prone to error due to subjective judgement. These shortcomings can be addressed by automatic term recognition (ATR), the process of annotating an electronic document with a set of terms extracted from the document [53]. Here, we emphasise that ATR refers to the computer-based extraction of terms from a domain-specific corpus as opposed to merely matching the corpus against a dictionary of terms [54]. It has been suggested that scientific corpora can be used as reliable sources for terminology construction exploiting [8]:

• the growing number of electronic corpora,

• efficient NLP tools (such as part-of-speech taggers, parsers, etc.),

• linguistically and/or statistically based ATR procedures, and

• the fact that domain experts often use terms that have not been standardised, and as such are not included into standardised dictionaries.

The lack of terminological standards is especially apparent in the rapidly expanding domain of metabolomics, where there is no exact consensus on what constitutes a metabolite name although naming conventions do exist for some entities, e.g. the Chemical Entities of Biological Interest (ChEBI) dictionary that is emerging for small molecules [55]. Still, these are only guidelines and as such do not impose restrictions on domain experts.

Manual term recognition is performed by relying on conceptual knowledge, i.e. humans identify terms by relating them to the corresponding concepts. It is currently not feasible to implement an ATR approach following such a paradigm due to the lack of appropriate knowledge representation systems and the difficulty of automatically performing “intelligent” tasks. For these reasons, ATR approaches resort to other types of knowledge that can provide clues about the terminological status of a given natural language clause [56]. Generally, the knowledge used for ATR may involve two types of information:

• internal: morphological, syntactic, semantic and/or statistical knowledge about terms and/or their constituents (nested terms, words, morphemes), and

• external: linguistic and/or statistical knowledge regarding the term context, together with the knowledge contained in external resources, such as electronic dictionaries, ontologies, corpora, etc.

ATR methods typically combine two approaches: linguistic (or symbolic) and statistical (or numeric) [51]. Linguistic approaches to ATR usually involve pattern matching to recognise candidate terms by checking if their internal structure conforms to a predefined set of morpho-syntactic rules. Statistical methods rely on at least one of the following hypotheses regarding the term usage [7]:

• specificity: terms are likely to be confined to a single or few domains,

• absolute frequency: terms tend to appear frequently in their domain, and

• relative frequency: terms tend to appear more frequently in their domain than in general.

Statistical approaches are prone to extracting not only terms, but also other types of collocations (sequences of words co-occurring more frequently than would be expected by chance) [57]: functional, semantic, thematic and others, e.g. “…*to play an important role in*…”. This problem is typically remedied by employing linguistic filters to extract candidate terms from a corpus, which are then ranked using statistical methods.

In this work, we utilised the C-value method [58], publicly accessible at [59] to the TM community via a web service. It first applies syntactic pattern matching to select term candidates, e.g. noun phrases having the structure described by the following regular expression:

$${\left(ADJ|N\right)}^{+}|\;\left({\left(ADJ|N\right)}^{*}\;\left[N\;PREP\right]\;{\left(ADJ|N\right)}^{*}\right)\;N$$

where *ADJ*, *N* and *PREP* denote adjective, noun and preposition respectively. The C-value of each candidate term *t* is then calculated as:

$$C-value\left(t\right)=\{\begin{array}{ll} \ln|t|\cdot f\left(t\right) & ,if\;S\left(t\right)=\emptyset \\ \ln|t|\cdot \left(f\left(t\right)-\frac{1}{\left|S\left(t\right)\right|}\sum_{s\in S\left(t\right)}f\left(s\right)\right) & ,if\;S\left(t\right)\neq \emptyset \end{array}$$

where |*t*| is the length of *t* in words, *f*(*t*) is *t*'s frequency of occurrence and *S*(*t*) is the set of other term candidates containing *t* as a sub-phrase. All candidates whose C-value exceeds a certain threshold are proposed as domain-specific terms by this method. The threshold chosen will affect the performance of ATR in terms of precision and recall, which are calculated as *P* = *A* / (*A* + *B*) and *R* = *A* / (*A* + *C*), where *A* is the number of true positives (correctly recognised terms), *B* is the number of false positives (phrases incorrectly recognised as terms) and *C* is the number of false negatives (non-recognised terms). Higher thresholds will typically result in higher precision and lower recall, and vice versa, lower thresholds will increase the recall at the expense of precision. In general, a threshold used should be corpus-specific (e.g. the average C-value found in the given corpus), as the C-value of each term candidate also depends on the corpus.

By its definition, the C-value method favours longer and more frequent phrases that are not typically nested within a relatively small set of other phrases. Obviously, the C-value method relies primarily on the frequency of term usage and their general syntactic properties rather than exploiting orthographic, morphological and lexical features of specific named entities. For example, while protein names may vary significantly between authors, some general characteristics still apply [60,61]:

• distinctive orthographic characteristics of protein names such as capital letters, digits, special characters (e.g. *p54SAP kinase*),

• keywords (e.g. *protein*, *receptor*, etc.) describing the protein function in multi-word protein names (e.g. *Ras GTPase-activating protein*, *EGF receptor*), and

• morphological principles for naming proteins, such as highly abundant affixes -*ase*, -*in*, etc. (e.g. *hexokinase*, *haemoglobin*).

Opting for a similar named entity recognition approach would significantly increase the time and cost of developing CV term acquisition methods, as these would have to be re-implemented for specific domains. Moreover, the type of terms sought may not necessarily exhibit sufficiently discriminatory textual properties [32].

On the other hand, a generic ATR approach (such as the C-value method) can be manipulated to extract terms that are more likely to be of the required type by targeting only relevant documents, and within them specific sections potentially dense with terms of the given type. This can be followed by additional filtering of terms, known to be of different and not directly relevant semantic types to the ones needed, by using lexical resources of these terms where such resources exist. This issue of ATR targeting only relevant documents has been addressed by the IR module described in the previous section. A domain-specific corpora is produced as a result of IR by using either MeSH or CV terms in the search queries over collections of either abstracts or full-text articles in PubMed.

Further, it is particularly important to target only sections that are likely to contain terms relevant for an analytical technology as a preparation step for ATR in order to increase its precision. Therefore, when using full-text documents we reduce them to the *Materials and Methods* sections, which are recognised automatically utilising PMC's XML format in which articles are distributed. Once a domain-specific corpus is obtained, the C-value terms are extracted and further inspected to see if they include any terms known to belong to other sub-domains not directly related to the analytical technology under investigation, in which case they can be safely filtered out.

### Term filtering

Given the initially compiled CVs for NMR and GC, we automatically obtained terms loosely related to these two analytical techniques by applying IR to compile a technology-specific corpus, followed by ATR to extract a list of terms from the corpus in a way described in the preceding sub-sections. Manual inspection of the extracted terms revealed typical types of terms frequently co-occurring with the NMR- and GC-specific terms, namely those denoting substances, organisms, organs, conditions/diseases, etc., which are not of direct interest for the analytical technology *per se*. Examples of such terms automatically extracted by the C-value method are: *amino acid*, *linseed oil*, *pancreatic juice*, *blood glucose*, *cell wall*, *Halophilic bacterium*, *Streptomyces antibioticus*, *systemic hypertension*, *cervical dislocation*, etc. Unlike analytical techniques, many of which are relatively recent, some of these terminologies are relatively stable with respect to the number of new terms being introduced, e.g. Linnaean taxonomy [62] classifies living organisms in a systematic manner.

The Unified Medical Language System [63] is a multi-purpose resource merging information from over 100 biomedical source vocabularies developed for different purposes. By providing uniform access (including a web service) to terms belonging to various sub-domains of interest, UMLS aims to facilitate the development of information systems for text processing in biomedicine via a semi-formal representation of domain-specific knowledge in order to process, retrieve, integrate, and aggregate biomedical data and information contained in the relevant literature [64]. It currently contains 1.4 million concepts named by 7.2 million terms, organised into a hierarchy of 135 semantic types and interconnected by 54 different relations.

The following semantic types in the UMLS proved relevant to our problem of detecting technique-specific terms in a subtractive approach: *Organism*, *Anatomical Structure*, *Substance*, *Biological Function* and *Injury or Poisoning*. Given these semantic types as part of the input to the term filtering module (implemented as a Java application), the subsumed terms are automatically selected from the latest version of the UMLS thesaurus. Then, a simple pattern matching approach is applied to filter out these terms and their variations. For example, the filtering approach helped identify the following “outliers” amongst terms extracted by the C-value method: *experimental rat*, *bovine heart muscle*, *maternal blood sera specimen*, *farmworker pesticide exposure*, *arterial carbon dioxide tension*, etc., simply by matching the UMLS terms from the above mentioned classes (e.g. *rat*, *bovine*, *heart*, *muscle*, *blood*, *pesticide*, *carbon dioxide*, *tension*).

### Output

**Figure 3:**
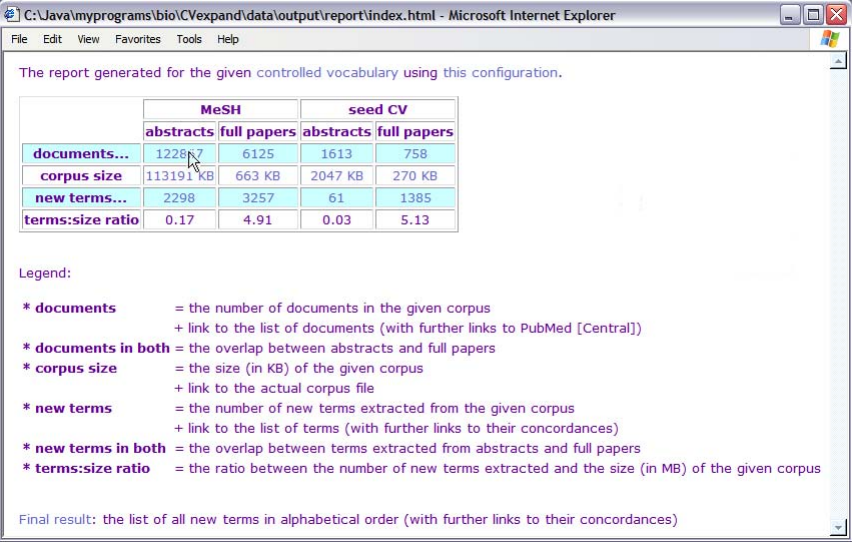
An HTML report summarising CV expansion results

**Figure 4:**
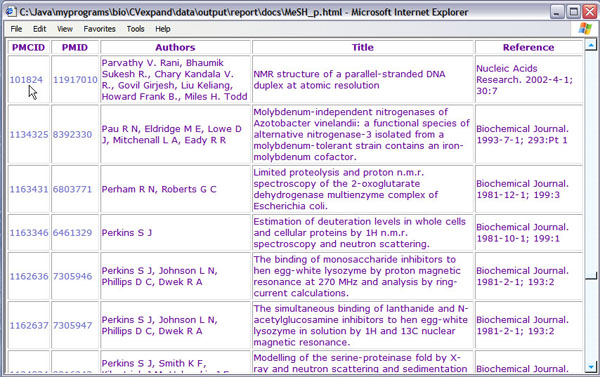
Citation details of the retrieved documents

**Figure 5:**
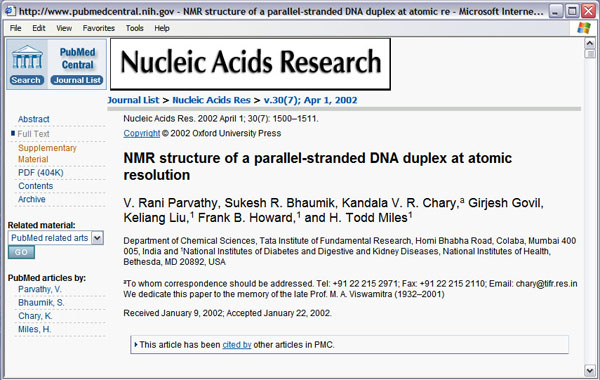
A full-text document retrieved from PMC

**Figure 6:**
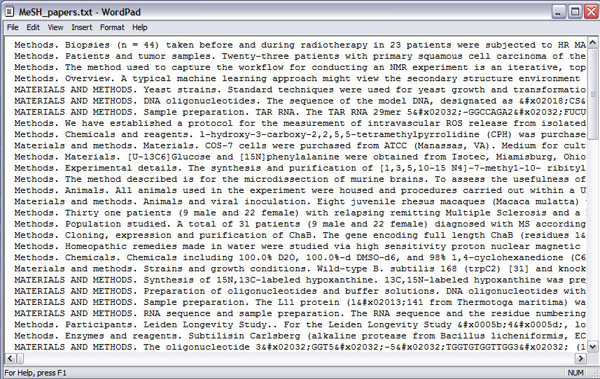
A corpus of “Materials and Methods” sections

We have described an integrative approach combining relatively generic software (e.g. Entrez for IR, C-value for ATR) and data resources (e.g. UMLS as a semantic network of biomedical terms) for the rapid development of a TM tool for automatic expansion of CVs as a practical alternative to tailor-made named entity recognition methods (see discussion above). An HTML report is generated as a result of the automated CV expansion (see Figure 3 for an example report generated for the NMR CV). The report summarises the output of each module described earlier, i.e.:

• the number of documents collected by the IR module with a link to the list of their citation details (see Figure 4) and cross-references to the actual documents in PubMed (see Figure 5)

• the size of the final text corpus with a link to the corresponding ASCII file (see Figure 6), and

• the number of new terms extracted by ATR with a link to the list of terms sorted by their C-values.

**Figure 7:**
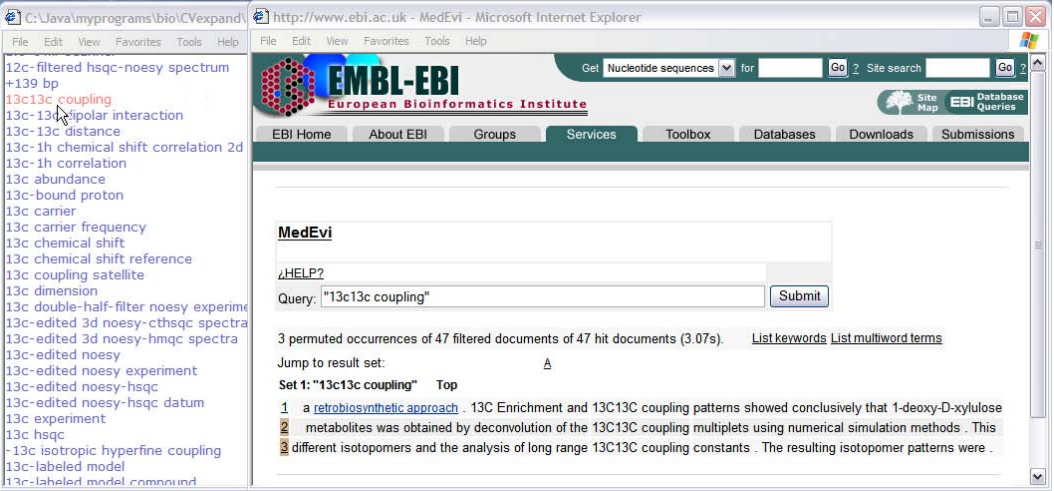
A list of automatically extracted terms with links to their concordances

Terms extracted from four different corpora are also amalgamated into a single, alphabetically ordered list (see Figure 7, left-hand side window). To aid the curation of automatically extracted terms and their incorporation into the CV, the context of a term can be obtained on-the-fly. The context should help the curator interpret the intended meaning of a term and provide clues useful for generating its textual definition. The context of a term rather than its definition may be more crucial for the association of a term with its correct meaning [65]. Terms sharing the same context are likely to have similar (or even the same) meaning [66]. Conversely, different contexts of the same term may point to the problem of term ambiguity (the same term denoting different concepts). Less drastically, the context may “deviate” the meaning of a term by emphasising only certain aspects of a term (e.g. insulin can be interpreted as both hormone and pharmacological substance). Bearing in mind the importance of contextual information in determining the correct meaning of a term and hence its position in a CV, we deployed a practical solution: all new terms reported are linked to MedEvi [67], a service providing local context (extracted from MEDLINE) for query terms [68]. Clicking on a term launches a query to MedEvi, which in turn returns the aligned concordance (words used in a context) lines together with some handy features such as lists of co-occurring keywords and terms (see Figure 7, right-hand side window).

## Results and discussion

We performed two case studies to evaluate the effectiveness of the proposed CV expansion approach using the two CVs for NMR and GC, which are currently under development as part of the MSI OWG activities. The initial CVs were compiled manually by the MSI OWG members, providing a total of 243 and 152 terms for NMR and GC respectively. In addition to these terms, we hand-picked the MeSH terms (*Magnetic Resonance Spectroscopy* and *Chromatography*, *Gas*) relevant for the techniques of interest by using the web-based MeSH browser. We used the given MeSH terms to retrieve documents from PubMed that have been manually annotated with these terms. A complementary IR approach was based on the search queries combining the CV terms: at least 3 and 7 matching terms for abstracts and full papers respectively.

Tables 1 and 2 provide the IR and ATR results. The top two rows refer to the IR approach used for collecting a corpus of relevant documents. The use of MeSH and CV terms to conduct searches over abstracts and full-text documents results in a total of four corpora, whose numerical properties are described in separate columns. The size of each corpus is given as the number of documents retrieved and its size in KBs (rows three and four). Although freely available for browsing, for most articles in PMC the publisher does not allow downloading of the text in XML format; neither does PMC allow bulk downloading in HTML format. Hence, we were able to process only a small number of full-text documents (the numbers in brackets refer to these papers). Total numbers of C-value terms extracted from each corpus are given in the bottom two rows, one referring to the total number of terms recognised by the C-value method and the other referring to the number of these terms remaining after applying the filtering approach based on the available knowledge about their semantic types.

**Table 1:** Term acquisition results for NMR

**IR**	**search terms**	**MeSH**		**CV**	

	**document type**	**abstracts**	**full papers**	**abstracts**	**full papers**
**corpus size**	**documents**	122,867	6,125 (141)	1,613	758 (29)

	**KBs**	113,191	663	2,047	270

**C-value terms**	**before filtering**	5,602	6,215	124	2,601

	**after filtering**	2,298	3,257	61	1,385

**Table 2:** Term acquisition results for GC

**IR**	**search terms**	**MeSH**		**CV**	

	**document type**	**abstracts**	**full papers**	**abstracts**	**full papers**
**corpus size**	**documents**	60,338	1,351 (79)	3,948	1,383 (58)

	**KBs**	42,418	68	3,012	97

**C-value terms**	**before filtering**	2,708	811	2,442	1,114

	**after filtering**	567	348	1,323	526

By amalgamating all filtered terms, a total of 5,699 and 2,612 new terms were acquired for NMR and GC respectively. The bottom rows in Tables 1 and 2 show their distribution across the four corpora. Note that the total number of new terms does not correspond to the sum of these numbers due to duplication of terms extracted from different corpora. Given a type of search terms (i.e. MeSH or CV terms), we compared the ATR results acquired from abstracts and those obtained from *Materials and Methods* sections of full-text articles. We determined that the overlap between the terms extracted from abstracts and those from the body of full-text articles was 2% on average. By further contrasting the results acquired from abstracts and full-text articles, we determined the average ratio between the number of acquired technology-specific terms and the corpus size was 16.25 for full-text articles and only 0.13 for abstracts. This comparison confirms that the *Materials and Methods* sections represent a significant source of technology-specific terms and also emphasises the benefits that can result from making full-text articles available to TM applications for the benefits of the overall biomedical community.

**Figure 8:**
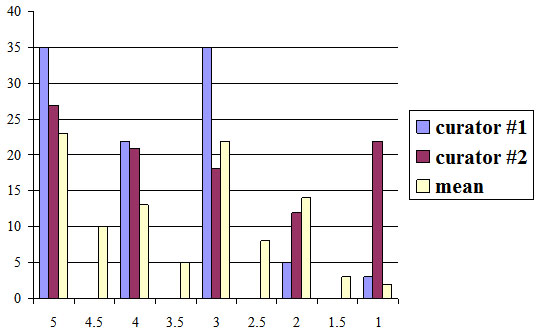
Distribution of evaluation scores for NMR

**Figure 9:**
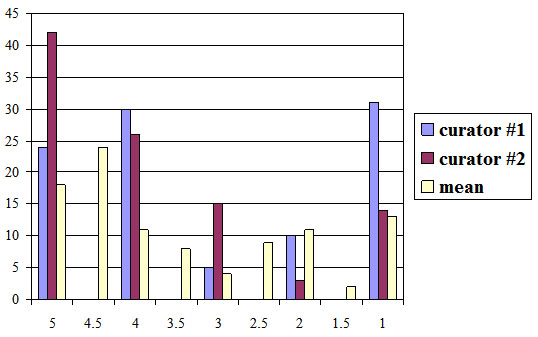
Distribution of evaluation scores for GC

The preliminary results are available at [14], where the potential CV terms are accessible to the metabolomics community for comments and curation. The official version of the NMR CV has been made publicly available at [22] as part of the NMR ontology. We have to note that the integration of new terms into the MSI CVs has only just started and a full evaluation can only be published later on the web pages. Nevertheless, we performed a preliminary evaluation using the following setup. For each case study, we selected a test set of 100 terms chosen randomly from the resulting set of candidate CV terms. Each test set was evaluated independently by two domain experts. Each term from the test sets was scored from 1 to 5 reflecting an expert opinion about the degree to which the term in question is related to the technology described by the CV: 1 – no, definitely; 2 – no, probably; 3 – don't know / not sure; 4 – yes, probably; 5 – yes, definitely. The detailed evaluation results are given in Additional File 1, where a reader can find the score given to each term by each of the curators. We also provide a mean score for each evaluated term and we measure the agreement between the curators by giving the score difference for each of the terms. The mean and median values for all scores are summarised in Tables 3 and 4. In both cases, the mean value of the average score was around 3.5 with the average difference in scores given by two curators not being greater than one. The distribution of the scores is shown in Figures 8 and 9. From these results we extract the fact that in the case of NMR 51 terms were deemed relevant (having an average score greater than 3), 22 terms were undecided (having an average score of 3) and 27 terms were deemed irrelevant (having an average score less than 3). Similarly, in the case of GC we obtained 61 positive examples, 35 negative ones and 4 undecided. By projecting these numbers to the total of 5,699 candidate NMR terms extracted, we estimate the numbers of relevant, undecided and irrelevant terms to be 2,906, 1254 and 1539 respectively. For the total of 2,612 candidate GC terms, it is projected that 1,593 will be relevant, 104 undecided and 914 irrelevant. By including ≈2,900 positive examples into the NMR CV (initially containing 243 terms) and ≈1,600 new terms into the GC CV (initially containing 152 terms), both CVs can be effectively expanded by more than ten times the original size simply by curating terms as opposed to the process of CV term collection using interviewing techniques and reading the relevant literature.

**Table 3:** Evaluation of term acquisition results for NMR

**score**	**by curator #1**	**by curator #2**	**mean between #1 & #2**	**difference between #1 & #2**
**mean**	3.81	3.19	3.5	0.88
**median**	4	3	3.5	1

**Table 4:** Evaluation of term acquisition results for GC

**score**	**by curator #1**	**by curator #2**	**mean between #1 & #2**	**difference between #1 & #2**
**mean**	3.06	3.79	3.425	0.93
**median**	4	4	4	1

In addition to the preliminary quantitative evaluation, we also provide some qualitative remarks about our approach TM approach to CV expansion, which will be taken into account in order to improve the functionality of the tool. Some of the extracted terms were “incomplete”. For example, the term *comparative NMR* as found in the result list lacks the headword to be of sufficient understandability and to get inserted into a CV, e.g. as its concordance () reveals this term should be *comparative NMR analysis* or *comparative NMR study*. This is due to the term variation phenomenon when the same concept is designated by more than one term. When such term candidates are processed separately, their C-values are distributed across different variants providing separate frequencies for individual variants instead of a single frequency unifying all of the variants. Hence, in order to make the most of the statistical part of the C-value method, term candidates need to be normalised prior to statistical analysis [69].

Further, the CV expansion process can be helped by a different way of presenting the resulting terms. Having the candidate terms clustered according to their head noun phrases (e.g. *experiment*, *assay*, *spectrum*, *chemical shift*) would facilitate term integration and hierarchical structuring of the CV.

## Conclusions

We described an integrative approach combining relatively generic, public software and data resources for time- and cost-effective development of a TM tool to aid the expansion of CVs across various domains. This should serve as a practical alternative to both manual term collection and tailor-made named entity recognition methods. The software makes use of web services to access three key resources:

• Entrez for IR,

• C-value for ATR, and

• UMLS as a semantic network of biomedical terms.

It is disseminated under an open-source licence. Originally developed to the specification of the MSI OWG, it is still generic enough to be applied for the expansion of other CVs in biomedicine simply by changing the input parameters:

• the initially compiled CV,

• the MeSH terms that reflect the domain of the CV, and

• the UMLS semantic types of terms indirectly related to those covered by the CV.

The output terms are presented to the user in HTML format so they can be inspected through a web browser, in which the context of each term as used in the scientific literature can be explored through the hyperlinked MedEvi service (a web-based search tool for the MEDLINE corpus) in an effort to aid the curation of the potential CV terms.

## Availability and requirements

Project name: CVexpand

Project home page: 

Operating system(s): Platform independent

Programming language: Java (version 1.6)

Other requirements: Access to SQL database

License: Academic Free License v3.0

Any restrictions to use by non-academics: None

## List of abbreviations used

ATR automatic term recognition

CV controlled vocabulary

DB database

GC gas chromatography

GC-MS gas chromatography – mass spectrometry

HUPO human proteome organization

HTML hypertext markup language

IR information retrieval

JDBC Java database connectivity

MEDLINE medical literature analysis and retrieval system online

MeSH medical subject headings

MGED microarray gene expression data society

MS mass spectrometry

MSI metabolomics standards initiative

NMR nuclear magnetic resonance

OBI ontology for biomedical investigations

OBO open biomedical ontologies

OWG ontology working group

PSI proteomics standards initiative

PMC PubMed Central

SQL structured query language

TM text mining

UMLS unified medical language system

XML extended markup language

## Competing interests

The authors declare that they have no competing interests.

## Authors' contributions

IS designed and implemented the text mining application and drafted the manuscript. DS provided the initial data, evaluated the results and helped to draft the manuscript. SAS conceived the overall study and participated in its design and coordination. DRS participated in the design and coordination of the text mining aspects of the study. DBK provided his expertise in metabolomics to help evaluate the results. NP supervised the bioinformatics integration aspects. MSI OWG members participated in provision of the data, discussions and evaluation. All authors read and approved the final manuscript.

## Supplementary Material

Additional File 1 Evaluation results: each test set was evaluated independently by two domain experts. Each term from the test sets was scored from 1 to 5 reflecting an expert opinion about the degree to which the term in question is related to the technology described by the CV: 1 – no, definitely; 2 – no, probably; 3 – don't know / not sure; 4 – yes, probably; 5 – yes, definitely. Click here for file
